# The pentose phosphate pathway regulates chronic neuroinflammation and dopaminergic neurodegeneration

**DOI:** 10.1186/s12974-019-1659-1

**Published:** 2019-12-05

**Authors:** Dezhen Tu, Yun Gao, Ru Yang, Tian Guan, Jau-Shyong Hong, Hui-Ming Gao

**Affiliations:** 1 MOE Key Laboratory of Model Animal for Disease Study, Model Animal Research Center, Institute for Brain Sciences, Nanjing University, 12 Xuefu Road, Nanjing, 210061 Jiangsu Province China; 2 Neurobiology Laboratory, National Institute of Environmental Health Sciences/National Institutes of Health, Research Triangle Park, Durham, NC 27709 USA

**Keywords:** Microglia, Neurodegeneration, Neuroinflammation, Pentose phosphate pathway, Glucose-6-phosphate dehydrogenase, NADPH oxidase, Oxidative stress, Metabolic disruption

## Abstract

**Background:**

Metabolic dysfunction and neuroinflammation are increasingly implicated in Parkinson’s disease (PD). The pentose phosphate pathway (PPP, a metabolic pathway parallel to glycolysis) converts glucose-6-phosphate into pentoses and generates ribose-5-phosphate and NADPH thereby governing anabolic biosynthesis and redox homeostasis. Brains and immune cells display high activity of glucose-6-phosphate dehydrogenase (G6PD), the rate-limiting enzyme of the PPP. A postmortem study reveals dysregulation of G6PD enzyme in brains of PD patients. However, spatial and temporal changes in activity/expression of G6PD in PD remain undetermined. More importantly, it is unclear how dysfunction of G6PD and the PPP affects neuroinflammation and neurodegeneration in PD.

**Methods:**

We examined expression/activity of G6PD and its association with microglial activation and dopaminergic neurodegeneration in multiple chronic PD models generated by an intranigral/intraperitoneal injection of LPS, daily subcutaneous injection of 1-methyl-4-phenyl-1,2,3,6-tetrahydropyridine (MPTP) for 6 days, or transgenic expression of A53T α-synuclein. Primary microglia were transfected with G6PD siRNAs and treated with lipopolysaccharide (LPS) to examine effects of G6PD knockdown on microglial activation and death of co-cultured neurons. LPS alone or with G6PD inhibitor(s) was administrated to mouse substantia nigra or midbrain neuron-glia cultures. While histological and biochemical analyses were conducted to examine microglial activation and dopaminergic neurodegeneration in vitro and in vivo, rotarod behavior test was performed to evaluate locomotor impairment in mice.

**Results:**

Expression and activity of G6PD were elevated in LPS-treated midbrain neuron-glia cultures (an in vitro PD model) and the substantia nigra of four in vivo PD models. Such elevation was positively associated with microglial activation and dopaminergic neurodegeneration. Furthermore, inhibition of G6PD by 6-aminonicotinamide and dehydroepiandrosterone and knockdown of microglial G6PD attenuated LPS-elicited chronic dopaminergic neurodegeneration. Mechanistically, microglia with elevated G6PD activity/expression produced excessive NADPH and provided abundant substrate to over-activated NADPH oxidase (NOX2) leading to production of excessive reactive oxygen species (ROS). Knockdown and inhibition of G6PD ameliorated LPS-triggered production of ROS and activation of NF-кB thereby dampening microglial activation.

**Conclusions:**

Our findings indicated that G6PD-mediated PPP dysfunction and neuroinflammation exacerbated each other mediating chronic dopaminergic neurodegeneration and locomotor impairment. Insight into metabolic-inflammatory interface suggests that G6PD and NOX2 are potential therapeutic targets for PD.

## Background

Parkinson’s disease (PD), the most common age-related neurodegenerative movement disorder, is characterized by progressive loss of dopamine (DA) neurons in the substantia nigra (SN) [1]. Unclear etiology, delayed diagnosis, no effective treatment, and clinical trial failures reinforce the need to identify new targets for drug discovery for PD treatment. Recently, emerging evidence has uncovered important roles of metabolic and inflammatory dysregulation in PD pathogenesis [2–5]. Neuroinflammation is a complex cascade of self-defensive response to injurious stimuli in the central nervous system (CNS). However, substantial evidence has demonstrated important contribution of neuroinflammation to PD pathogenesis. For instance, DNA polymorphisms or variations in multiple inflammatory cytokines and in the HLA (human leukocyte antigen) region that contains many immune-related genes are associated with increased risk for PD [6, 7]. The SN of PD patients and animal models showed microglial activation and accumulation of inflammatory mediators [5, 8, 9]. Moreover, inhibition of neuroinflammation correlates with less neuronal impairment in various PD models [10–12]. Therefore, to discern mechanism(s) flipping neuroinflammation from a beneficial physiological response to a chronic neurodegenerative one is urgent and of paramount importance.

Recently, increasing preclinical and clinical evidence has revealed glucose metabolism disturbance in PD [2, 13–15]. Interestingly, several recent studies have shown dysregulation of the pentose phosphate pathway (PPP, a metabolic pathway parallel to glycolysis) in PD [2, 13]. The PPP mainly coordinates anabolic biosynthesis and redox homeostasis by controlling its intracellular products ribose-5-phosphate (the biosynthetic precursor of nucleotides) and NADPH rather than provides energy supply [16]. Glucose-6-phosphate dehydrogenase (G6PD), the rate-limiting enzyme of the PPP, is a ubiquitous enzyme, but its expression and activity vary over a 10-fold range, being lowest in the skeletal muscle and highest in the brain [17, 18]. It has been reported that neurons preferentially metabolize glucose via the PPP [19]. Earlier measurement of G6PD in red blood cells in PD patients has yielded conflicting results claiming reduced or unaltered activity [20, 21]. A postmortem study of PD brains has detected an increase in NADPH production in the putamen (a brain region affected in PD) of late-stage cases; unexpectedly, the putamen of early-stage PD and the cerebellum of early- and late-stage PD display a reduction in G6PD [13]. Up to date, neither NADPH nor G6PD in the SN (the major disease region in PD) has been examined in patients or animal models of PD. Nevertheless, available evidence reveals dysregulation of the G6PD and the PPP in PD. Their possible pathogenic roles in PD warrant further investigation.

Oxidative stress is a major component and connector of metabolic disruption and neuroinflammation and serves as a key pathologic factor in neurodegenerative diseases including PD [2, 3, 10, 22]. NADPH generated in the PPP is a cofactor and a reducing agent used in anabolic reactions (e.g. synthesis of glutathione, lipid, and nucleic acid). On the other hand, under catalysis by activated NADPH oxidase (NOX2), NADPH provides electron to molecular oxygen to generate the free-radical superoxide. Over-activated NOX2 is a major source of oxidative stress under inflammatory condition and has been implicated as a novel therapeutic target for neurodegenerative diseases [10]. High energy requirement, high glucose consumption, adequate production of reactive oxygen species (ROS), low antioxidant defense, abundant oxidation-sensitive lipids, and a restricted renewal and regenerative capacity of neurons render the brain extremely susceptible to glucose metabolism disruption, oxidative insults, and inflammatory destruction. Therefore, it is important to investigate possible causal roles of PPP-mediated metabolism disturbance and neuroinflammation in PD. In the present study, we provided the first evidence that G6PD-mediated metabolic dysfunction in the PPP in brain microglia and neuroinflammation exacerbated each other inducing chronic neurodegeneration and locomotor impairment, thus identifying a mechanistic basis for chronic PD progression.

## Materials and methods

### Animals

All animals were treated in strict accordance with the National Institutes of Health (Bethesda, MD, USA) Guide for Humane Care and Use of Laboratory Animal. All efforts were made to minimize the number of animals and their suffering. Both timed-pregnant Fisher 344 rats and C57BL/6 J mice were obtained from the Jackson Laboratory (Bar Harbor, ME). The intranigral and intraperitoneal injection of lipopolysaccharide (LPS, 0111:B4) and daily subcutaneous injection of 1-methyl-4-phenyl-1,2,3,6-tetrahydropyridine (MPTP, 15 mg/kg) for 6 consecutive days were performed as previously described [23, 24]. At desired time points after the injection, mice were anesthetized with fatal plus and then transcardially perfused with ice-cold PBS, followed by rapid freezing in liquid nitrogen or perfusion with 4% PBS-buffered paraformaldehyde. Fixed and frozen brains were used for immunohistochemistry and western blotting assay respectively.

### Cell cultures

#### Primary neuronal and glial cultures

Mesencephalic neuron-glia cultures were prepared from the ventral mesencephalon of embryonic day 14 ± 0.5 Fischer 334 rats. Cultures were maintained in minimum essential medium (MEM) supplemented with 10% heat-inactivated fetal bovine serum (FBS) and 10% heat-inactivated horse serum (HS), 1 g/L-glucose, 2 mM L-glutamine, 1 mM sodium pyruvate, and 0.1 mM nonessential amino acids. Seven days after seeding, the cultures were either treated with vehicle or 10 ng/mL LPS with or without pretreatment with various reagents as specified in the figure legend. This concentration of LPS was used in all in vitro studies. At the time of treatment, the neuron-glia cultures were made up of ~ 12% microglia, 48% astrocytes, and 40% neurons of which 2.8–3.8% were tyrosine hydroxylase (TH)-immunoreactive (IR) neurons [25]. Rat neuron-glia cultures were used to examine effects of pharmacological inhibition of G6PD.

#### Mixed-glia cultures and microglia-enriched cultures

Mixed-glia cultures were prepared from whole brains of postnatal day 1 mice. Disassociated brain cells were seeded into 96-well plates (10^5^ cells/well) or 175 cm^2^ flasks (3 brains/flask) and maintained in DMEM/F-12 supplemented with 10% FBS, 2 mM L-glutamine, 1 mM sodium pyruvate, and 0.1 mM nonessential amino acids. The medium was changed every 3 days. When reaching confluence at 11–12 days after seeding, the cultures contained ~ 80% glial fibrillary acidic protein (GFAP)-IR astrocytes and ~ 20% ionized calcium-binding adapter molecule 1 (Iba1)-IR microglia and were used for treatment or preparation of microglia-enriched cultures. Microglia were separated from astrocytes by shaking the flasks containing confluent mixed-glia cultures for 1 h at 150 rpm. The purity of microglia-enriched cultures was greater than 98%, as determined by immunostaining for Iba1 and GFAP [25].

#### Reconstituted cell cultures

Mouse microglia were directly plated on top of the existing mouse neuron-astrocyte cultures [25]. Briefly, leucine-leucine methyl ester (LME; 1.5 mM; Sigma-Aldrich) was added to mesencephalic neuron-glia cultures 2 days after cell seeding to deplete microglia. Three days later, the cultures were changed back to fresh medium without LME. Six days after the initial seeding, the neuron-astrocyte cultures contained about ~ 54% astrocytes, 45% neurons, and less than 1% microglia as determined by immunostaining for GFAP, neuron-specific nuclear protein (Neu-N; 1:4000; Chemicon, Temecula, CA), and Iba1. Highly enriched microglia that were transfected with G6PD siRNAs or scramble siRNA for 30 h were added to the existing neuron-astrocyte cultures. The reconstituted cultures were treated with LPS or vehicle 12 h later. Five days after LPS/vehicle treatment, degeneration of DA neurons was assessed.

### Immunohistochemistry and immunocytochemistry

Immunohistochemistry and immunocytochemistry were performed as described previously [25] with antibodies specific for G6PD (1:2500; Abcam), TH (1:5000; Millipore), or Iba1 (1:5000; Wako Pure Chemicals). Briefly, paraformaldehyde-fixed floating brain sections or cell cultures were blocked with appropriate normal serum followed by incubation overnight at 4 °C with primary antibodies. After incubation with an appropriate biotinylated secondary antibody and then the Vectastain ABC reagents (Vector Laboratory, Burlingame, CA, USA), the bound complex was visualized by color development with 3,3′-diaminobenzidine. Images were recorded with a CCD camera and the ZEN 2.3 lite software (Molecular Devices). The number of TH-IR neurons in neuron-glia cultures and the SN of fourteen evenly spaced brain sections from a series of 48 sections that covered the entire SN was counted by two individuals blind to the treatment.

### Immunofluorescence

Mouse brain sections and cell cultures grown in glass chambers were used for double-labeled immunofluorescence. G6PD antibody (1:2500; Abcam), in combination with an antibody specific for CD11b (1:500; Bio-Rad Laboratories), TH (1:2000; Millipore), Neu-N (1:4000; Chemicon, Temecula, CA), or GFAP (1:1000; LifeSpan BioScience), was used as indicated in the figure legend. Phosphorylated p65 antibody (1:2000; Cell signaling) was used to detect NF-κB activation in microglia-enriched cultures. Brain sections or cell cultures were then incubated with Alexa-488 (green) and/or Alexa-594 (red) conjugated secondary antibodies (1:1000; Invitrogen). All fluorescent images were obtained with a Zeiss LSM 780 or 880 NLO laser scanning confocal microscope.

### Western blotting assay

Whole cell proteins were extracted from cultured cells by using radioimmunoprecipitation assay (RIPA) lysis buffer (50 mM Tris-HCl, pH 8.0, 150 mM NaCl, 5 mM EDTA, 1% NP-40, 0.5% sodium deoxycholate, 0.1% SDS, and protease inhibitor cocktail). Protein concentrations were determined by using the biocinchoninic acid assay (BCA, ThermoFisher). Protein samples were resolved on NuPAGE 4-12% Bis-Tris gels (Life technologies), and immunoblot analyses were performed using antibodies against G6PD (1:5000; Abcam), TH (1:5000; Millipore), or Iba-1 (1:2500; Wako Pure Chemicals). An antibody against β-actin or GAPDH (1:5000; Cell Signaling Technology) was included to monitor loading errors.

### Nuclear and cytosolic fractionation

Nuclear and cytosolic proteins were extracted at 4 °C from primary microglia using the NE-PER™ Nuclear and Cytoplasmic Extraction Reagents kit (ThermoFisher) following the manufacturer’s instructions. The volume ratio of CER I, CER II, and NER was 200:11:100. Microglia pellets were fully suspended in CER I with vigorous vortex at the highest speed and incubated on ice for 10 min. Ice-cold CER II was added into the mixture, followed by vigorous vortex for 5 s and incubation on ice for 1 min. The cell lysis was centrifuged for 5 min at maximum speed in a microcentrifuge (~ 13,000×*g*). The supernatant (cytoplasmic extract) was immediately transferred to a prechilled tube and placed on ice before use or storage. After rinsed twice with CER I to avoid cytosolic protein contamination, nuclei were suspended in ice-cold NER with vigorous vortex for 15 s and incubated on ice for 40 min with continue vortex for 15 s every 10 min. Nuclear extracts, collected by centrifuge at maximum speed (~ 13,000×*g*) in a microcentrifuge for 10 min, were immediately transferred to a prechilled tube and placed on ice before use or storage. Nuclear and cytosolic extracts were used to examine NF-κB activation by Western blotting assay using antibodies against phosphorylated or total NF-κB subunit p65 (1:2500, cell signaling). While anti-histone H3 antibody (1:2500, Abcam) was used to monitor loading errors of nuclear proteins, anti-GAPDH (1:5000, cell signaling) or anti-tubulin antibody (1:5000, ThermoFisher) was used to monitor loading errors of cytosolic proteins.

### High-affinity [^3^H]DA uptake assay

The uptake assay was performed as described [25]. Briefly, after being rinsed twice with warm Krebs-Ringer buffer (KRB; 16 mM sodium phosphate, 119 mM NaCl, 4.7 mM KCl, 1.8 mM CaCl_2_, 1.2 mM MgSO_4_, 1.3 mM EDTA, and 5.6 mM glucose; pH 7.4), neuron-glia cultures were incubated for 15 min at 37 °C with 1 μM [^3^H]DA (30 Ci/mmol, NEN, Boston, MA, USA) in KRB. After being washed three times with ice-cold KRB, cells were solubilized in 1 M NaOH, and radioactivity was counted with a liquid scintillation counter. Nonspecific uptake was determined in the additional presence of 20 μM GBR 12935 dihydrochloride (a DA transporter inhibitor; S9659, Sigma-Aldrich).

### Superoxide assay

The production of superoxide was determined by measuring the superoxide dismutase (SOD)-inhibitable reduction of tetrazolium salt WST-1, as described previously with modifications [26]. Primary microglia (1 × 10^5^ cells/well), grown overnight in clear 96-well plates, were pretreated with vehicle or G6PD inhibitors for 30 min or transfected with G6PD siRNAs or scramble siRNA for 30 h followed by treatment with LPS in phenol red-free medium. The cultures were incubated with WST-1 (1 mM) with or without 800 U/mL SOD for 30 min at 37 °C, and the absorbance at 450 nm was read with a SpectraMax Plus microplate spectrophotometer (Molecular Devices, Sunnyvale, CA).

### Detection of intracellular ROS

The production of intracellular ROS (iROS) was measured by the cell-permeable fluorescence probe chloromethyl-29, 79-dichlorodihydrofluorescein diacetate (CM-H_2_DCFDA; Cat# C6827; Invitrogen) or 2′,7′-Dichlorofluorescin Diacetate (H_2_DCFDA; Cat# 287810; Calbiochem) as described [27]. Briefly, mixed-glia cultures in black 96-well plates were pretreated with vehicle or G6PD inhibitors for 30 min followed by treatment with LPS for 6 h in phenol red-free medium. After incubated with 10 μM H_2_DCFDA for 1 h at 37 °C, the fluorescence density was read at 488 nm for excitation and 525 nm for emission using a SpectraMax Gemini XS fluorescence microplate reader (Molecular Devices). Alternatively, microglia-enriched cultures grown in glass chambers were transfected with G6PD siRNAs or scramble siRNA for 30 h or pretreated with vehicle/G6PD inhibitors for 30 min followed by treatment for 18 or 24 h with LPS as described in the figure legend. The cultures were stained with 10 μM CM-H_2_DCFDA or antibody against 4-hydroxynonenal (4-HNE; 1:500; Abcam). Brain sections were immune-stained for 3-nitrotyrosine (3-NT; 1:500; Abcam). Fluorescent images were collected using Zeiss LSM 780 or 880 confocal microscope and analyzed by using ImageJ software.

### Measurement of tumor necrosis factor-α

The level of tumor necrosis factor-α (TNFα) at 6 h after LPS treatment in the culture medium was measured with commercial ELISA kits (R&D Systems) following the manufacturer’s instructions.

### G6PD enzyme activity assay and NADPH measurement

Mouse mesencephalic neuron-glia cultures were treated with either vehicle or LPS. The activity of G6PD and the level of NADPH in cell lysis from the cultures were measured following the manufacturer’s instructions using Glucose 6 Phosphate Dehydrogenase Assay Kit (Colorimetric; ab102529; Abcam) and NADP/NADPH Assay Kit (ab65349; Abcam), respectively.

### Transfection with siRNA

Mouse microglia grown in 24-well/6-well plates were transfected with G6PD siRNAs (ThermoFisher, 4390843 and 4390771) or scramble siRNA for 6 h using Lipofectamine™ RNAiMAX transfection reagent (ThermoFisher, 13778) and antibiotic-free Opti-MEM® I reduced serum medium following the manufacturer’s instructions and previous publication [28]. The final concentration of siRNAs is 10 nM. The cultures were then changed to normal growth medium, and 24 h later, the knockdown efficiency was assessed by western blotting assay.

### Lactate dehydrogenase cytotoxicity assay

Measurement of extracellular lactate dehydrogenase (LDH) was performed using LDH cytotoxicity detection kit (Colorimetric, Takara) following the manufacturer’s instructions. Briefly, the reconstituted 2X LDH assay buffer (50 μL) and the supernatant (50 μL) were mixed by gentle shaking for 30 s and incubated at room temperature for 30 min. The reaction was stopped by adding 50 μL of 2 N HCl, and the absorbance value at 490 nm was read.

### MTT assay for cell viability

Thiazolyl blue tetrazolium bromide (MTT, 0.45 mg/mL, Sigma-Aldrich) was added to cultures treated with 6-aminonicotinamide (6-AN, Sigma-Aldrich) and dehydroepiandrosterone (DHEA, Sigma-Aldrich) for a desired period of time. After incubation for 2 h at 37 °C, cell medium was removed from the cultures. The precipitated formazan, a product of MTT by the action of mitochondrial dehydrogenases, was solubilized with dimethyl sulfoxide and quantified spectrophotometrically at 570 nm.

### Rotarod behavior test

The rotarod behavior test was performed on a Rotamex device (Columbus Instruments, Columbus, OH, USA). The start speed was 0.5 rpm, the acceleration rate was set to 0.5 rpm/10s, and the maximum speed was 50 rpm. The mice underwent three consecutive trials with 30-min interval. The latency time to fall was recorded and analyzed to evaluate the motor coordination of mice.

### Statistical analysis

All values are expressed as the mean ± SEM. Differences among means were analyzed by using one- or two-way ANOVA with treatment as the independent factors. When ANOVA showed significant differences, multiple comparisons between means were tested by Dunnett’s, Turkey, or Sidak’s multiple post hoc testing. In all analyses, the null hypothesis was rejected at the 0.05 level.

## Results

### Sustained high activity of the PPP in PD models

**Fig. 1:**
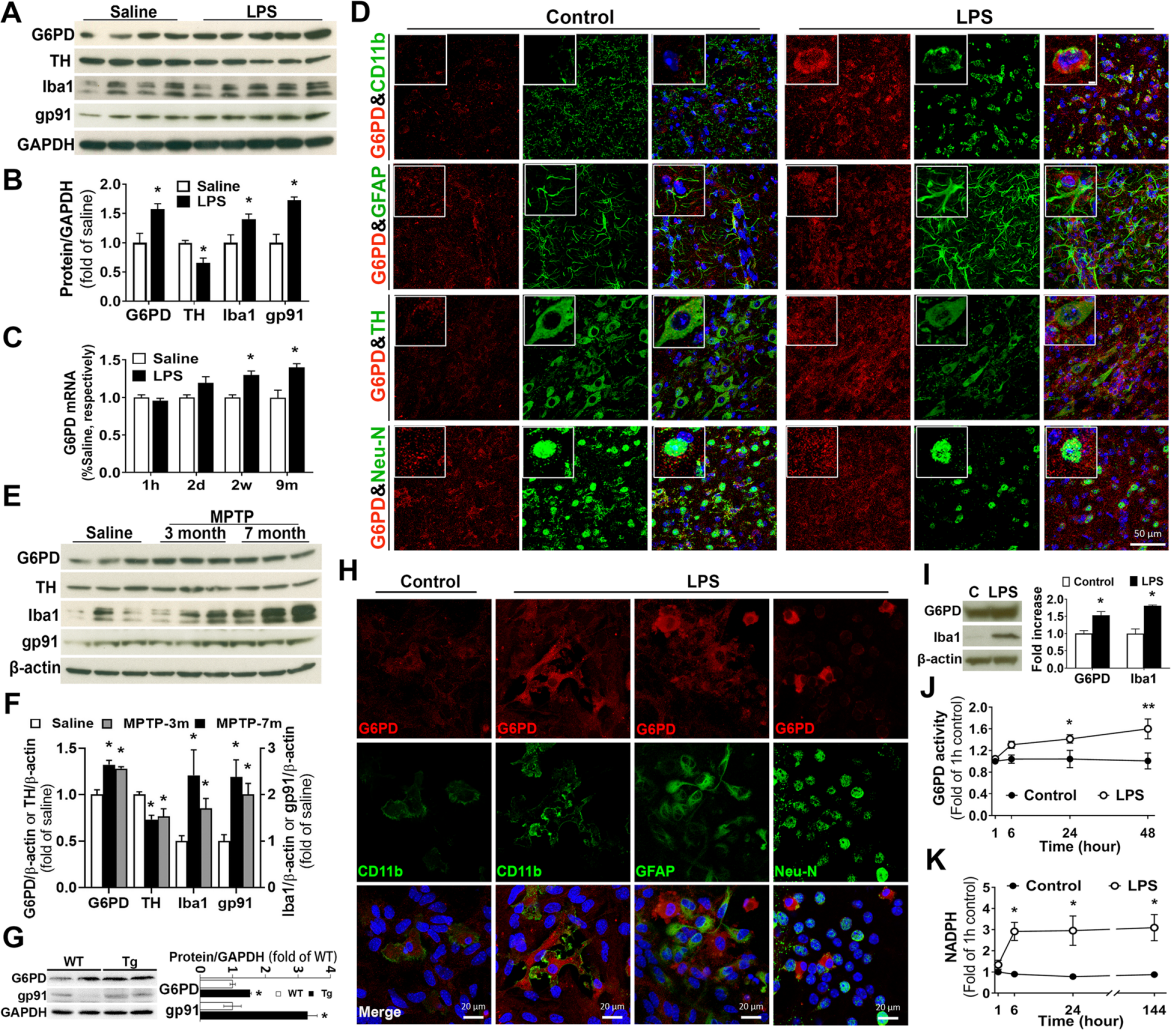
Increased expression and activity of the PPP in multiple PD models. **a** One year after an intraperitoneal injection of 5 mg/kg LPS, mouse midbrains displayed increased expression of G6PD, gp91^*phox*^, and Iba1 and decreased expression of TH. **b** The ratio of densitometry values of these proteins in **a** was analyzed and normalized to each responsive control. **c** The mRNA level of G6PD in the midbrain of mice with an intraperitoneal injection of saline or 5 mg/kg LPS. **d** Double-labeled immunofluorescence of G6PD (red) with microglial marker CD11b, astroglial marker GFAP, DA neuron maker TH, or neuronal marker Neu-N (green) in mouse SN displayed dramatic G6PD upregulation in microglia at 2 weeks after an intranigral injection of 2 μg LPS. Enlarged inserts in the top panel showed increased G6PD staining in microglia. **e**, **f** At 3 or 7 months after daily subcutaneous injection of MPTP (15 mg/kg for 6 days), mouse midbrains exhibited increased expression of G6PD, gp91^*phox*^, and Iba1 and reduced levels of TH. **g** One-year-old A53T α-synuclein transgenic mice revealed upregulation of G6PD and gp91^*phox*^ in midbrains compared with age-matched wild-type mice. **h** Double-labeled immunofluorescence of G6PD (red) with CD11b, GFAP, or Neu-N (green) in neuron-glia cultures treated with LPS (10 ng/mL) for 48 h showed occurrence of LPS-induced upregulation of G6PD in activated microglia but not neurons or astroglia. Double-stained images of G6PD and CD11b in vehicle-treated control cultures, neurons, or astroglia, which were negative for CD11b staining and positive for DAPI staining, showed weak G6PD staining. **i** Increased expression of G6PD and Iba1 in microglia-enriched cultures upon LPS treatment for 24 h. **j**, **k** Rat mesencephalic neuron-glia cultures treated with LPS showed high activity of G6PD (**j**) and increased production of NADPH (**k**) compared with vehicle-treated cultures. All images are representative of three independent experiments. Results are the mean ± SEM of three independent experiments (**b**, **c**, **f**, **g**, **i**–**k**). *p < 0.05 and **p < 0.01 compared with vehicle-treated controls (**b**, **c**, **f**, **i**–**k**). *p < 0.05 compared with wild-type littermates (**g**)

To investigate the role of the PPP in PD, we first detected expression and activity of G6PD, the rate-limiting enzyme of the PPP, in four in vivo mouse PD models generated by an intranigral or intraperitoneal injection of LPS, daily subcutaneous injection of MPTP for 6 days, or transgenic overexpression of A53T mutant α-synuclein. Firstly, 1 year after an intraperitoneal LPS injection, mouse midbrains showed increased levels of G6PD, gp91^*phox*^ (the catalytic subunit of NOX2), and Iba1 and a decreased level of TH, indicating sustained upregulation of G6PD, chronic neuroinflammation, and dopaminergic neurodegeneration (Fig. 1a, b). Moreover, the mRNA level of G6PD was significantly increased at 2 weeks or 9 months after LPS injection as compared with age-matched saline-injected controls (Fig. 1c). Secondly, at 2 weeks after an intranigral injection of LPS, we detected dramatic G6PD upregulation in the SN, and upregulated G6PD was mainly located in microglia, but not in astroglia or neurons (Fig. 1d). The LPS-injected SN also showed profound activation of microglia and astroglia as well as damages and loss of DA neurons compared with vehicle-injected SN (Fig. 1d). Thirdly, in a sub-acute MPTP model of PD with daily subcutaneous injection of MPTP for 6 days, mouse midbrains displayed sustained upregulation of G6PD, gp91^*phox*^, and Iba1 as well as reduction in TH level at 3 and 7 months after the last MPTP injection (Fig. 1e, f). Fourthly, midbrains of 1-year-old A53T mutant α-synuclein transgenic mice showed increased levels of G6PD and gp91^*phox*^ compared with age-matched wild-type mice (Fig. 1g).

Double-labeled immunofluorescence in neuron-glia cultures treated with LPS for 48 h showed dramatic G6PD upregulation in activated microglia but not neurons or astroglia. Double-stained images of G6PD and CD11b in vehicle-treated control cultures, CD11b^−^/DAPI^+^ neurons and astroglia, and CD11b^+^/DAPI^+^ microglia all showed weak G6PD staining (Fig. 1h). Western blotting assay and immunostaining detected a significant increase in the level of G6PD protein in microglia-enriched cultures treated with LPS for 24 h (Fig. 1i and Additional file 1: Figure S1) and mesencephalic neuron-glia cultures treated with LPS for 7 days (Additional file 1: Figure S1). We next measured the enzyme activity of G6PD and the level of NADPH (a major product of the PPP and a substrate of NOX2) in mesencephalic neuron-glia cultures treated with vehicle or LPS. LPS-treated cultures exhibited long-lasting increases in G6PD activity and NADPH level compared with vehicle-treated cultures (Fig. 1 j, k). Collectively, in multiple PD models, both microglial activation and loss of nigral DA neurons had positive correlation with increases in the expression/activity of G6PD and the production of NADPH (Fig. 1a, b, d–h). The concurrent upregulation of G6PD and NOX2 suggested that microglia with elevated G6PD activity generated excessive NADPH and provided abundant substrate to over-activated NOX2 thereby inducing oxidative stress, neuroinflammation, and neurodegeneration. Thus, metabolic disturbance in the PPP might be involved in chronic neuroinflammation and PD neurodegeneration.

### Knockdown or pharmacological inhibition of G6PD protected DA neurons from LPS-induced degeneration

**Fig. 2:**
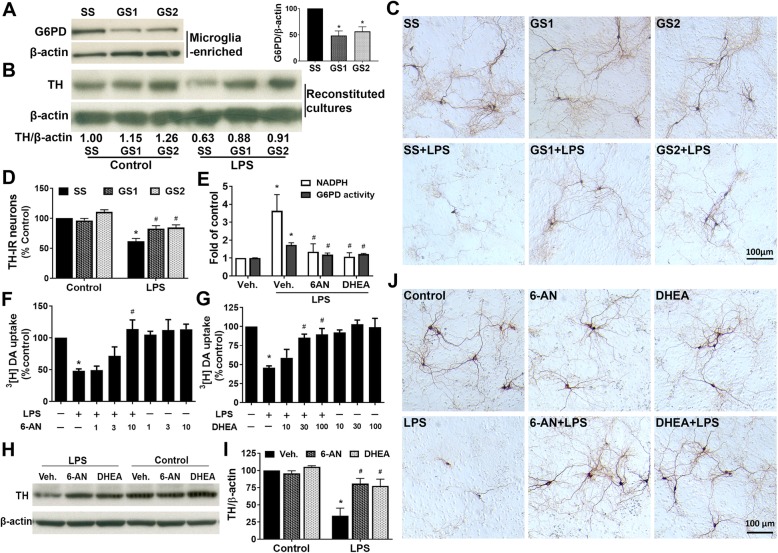
Knockdown or pharmacological inhibition of G6PD protected DA neurons from LPS-elicited inflammatory insults. **a** Primary mouse microglia-enriched cultures were transfected with 10 nM scramble RNA (SS) or G6PD siRNAs (GS1 and GS2) for 30 h before examining the knockdown efficiency by Western blotting assay. **b**–**d** The reconstituted cultures, which were prepared by adding mouse microglia (5 × 10^4^ microglia/well) with 30-h knockdown of G6PD by siRNAs onto mouse neuron-astrocyte layer, were treated with vehicle or LPS for 5–6 days. Western blotting assay (**b**), representative images (**c**), and cell counting (**d**) from three to four experiments revealed DA neuroprotection by knockdown of microglial G6PD. **e**–**j** Rat mesencephalic neuron-glia cultures were pretreated with vehicle, 6-AN, or DHEA for 30 min and treated with the vehicle or LPS. **e** Effects of 6-AN and DHEA on G6PD enzyme activity and NADPH levels at 2 days after LPS treatment. **f**–**j** Survival of DA neurons was determined by ^3^[H]DA uptake assay (**f**, **g**), western blotting assay (**h**, **i**), and representative images (**j**) at 7 days after LPS treatment. Results are mean ± SEM (**a**, **d**–**g**, **i**). *p < 0.05 compared with vehicle-treated controls. ^#^*p* < 0.05 compared with LPS-treated cultures

To explore whether there is a causal relationship between abnormal upregulation of G6PD and DA neurodegeneration, we utilized biological knockdown and pharmacological inhibition to decrease G6PD expression and activity. We prepared reconstituted cultures by adding microglia with 30-h knockdown of G6PD by siRNAs, which caused 45–53% decreases in G6PD protein levels, onto neuron-astrocyte layer (Fig. 2a). Western blotting results showed that LPS treatment for 6 days led to reduction in TH protein in reconstituted cultures containing microglia transfected with scramble siRNA (SS) but not G6PD siRNAs (GS1 or GS2). In another word, siRNA-mediated knockdown of microglial G6PD prevented LPS-induced reduction in TH protein levels (Fig. 2b). Immunocytochemical analysis demonstrated that LPS-induced degeneration of DA neurons, as shown by a significant loss of TH-IR perikaryon and destruction of TH-IR dendrites, was rescued by G6PD knockdown (Fig. 2c, d). Notably, following the LPS treatment in the cultures transfected with scramble siRNA, the number of TH-IR neurons was decreased, and the neurites of the remaining TH-IR neurons became shorter, lighter-stained, or even fragmented. G6PD knockdown protected DA neurons from such destruction (Fig. 2c, d).

We next determined how DA neurodegeneration was affected by inhibition of G6PD activity by 6-AN and DHEA that have been widely used to inhibit G6PD and the PPP to decrease intracellular NADPH levels [29–32]. DHEA is a steroid hormone, 6-AN has been reported to induce astroglial toxicity and neurotoxicity in specific nuclei in brainstem and spinal cord [33, 34], and no better G6PD inhibitor is available. We therefore used both 6-AN and DHEA in in vitro studies making the results sound and solid. We first confirmed that both 6-AN (10 μM) and DHEA (100 μM) blocked LPS-elicited increases in G6PD activity and NADPH production in rat mesencephalic neuron-glia cultures at 2 days after LPS treatment (Fig. 2e). LDH and MTT assays detected no cytotoxicity in rat neuron-glia cultures treated with 6-AN (10 μM) or DHEA (100 μM) for 1 or 7 days (data not shown). Pretreatment of mesencephalic neuron-glia cultures with 6-AN (1-10 μM) and DHEA (10-100 μM) significantly attenuated LPS-induced degeneration of DA neurons, which was determined by the functional assay of [^3^H]DA uptake, western blotting assay of TH protein, and immunocytochemical analysis of TH-IR neurons 7 days after LPS treatment (Fig. 2f–j). Collectively, both knockdown of G6PD expression and inhibition of G6PD activity prevented LPS-induced dopaminergic degeneration.

### Knockdown and pharmacological inhibition of G6PD attenuated LPS-elicited oxidative stress

**Fig. 3:**
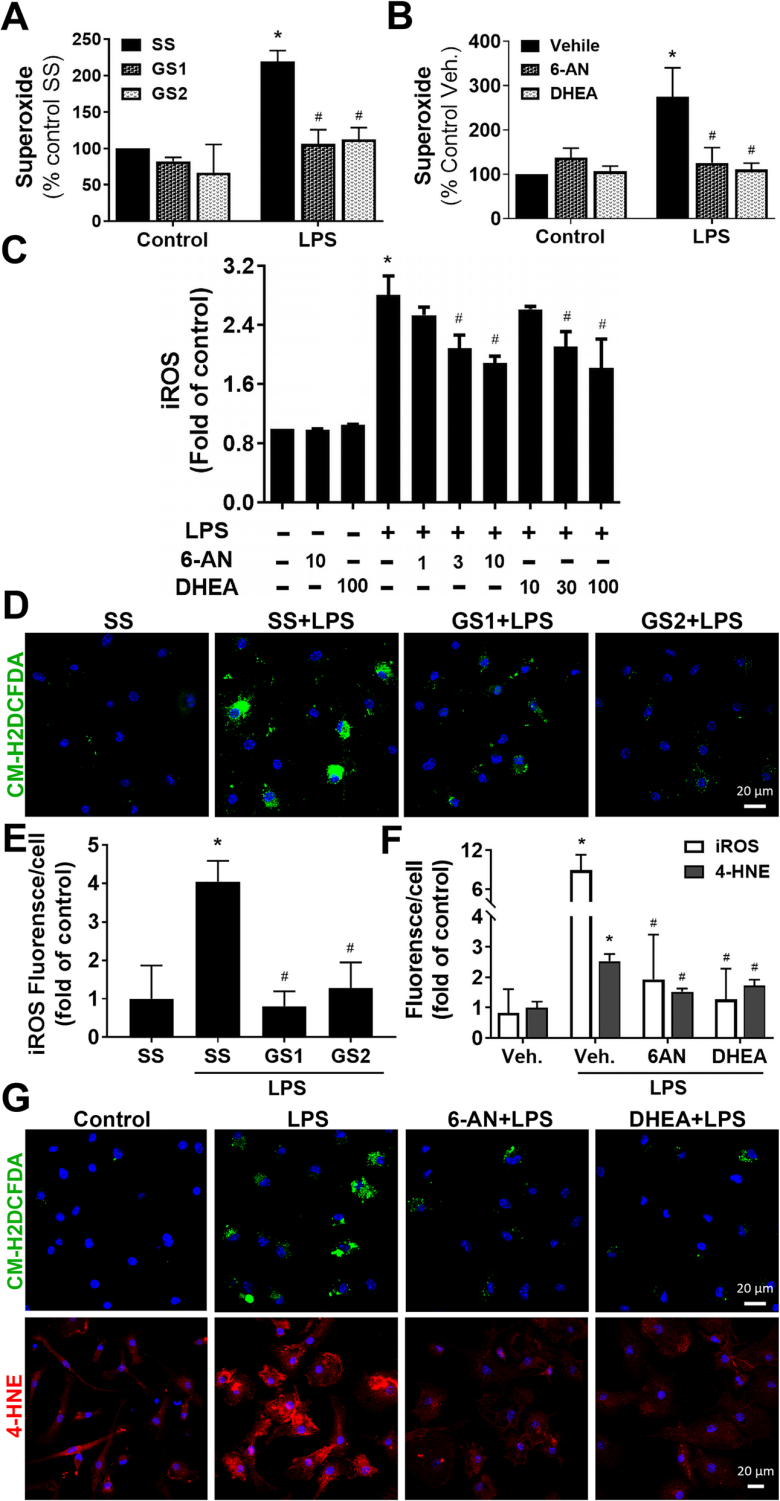
Knockdown or pharmacological inhibition of G6PD attenuated LPS-elicited ROS production. **a** Microglia-enriched cultures grown in culture chambers were transfected with 10 nM scramble siRNA (SS) or G6PD siRNAs (GS1 and GS2), and 30 h later, the cultures were treated with LPS. Superoxide production in microglia-enriched cultures was measured by SOD-inhibitable WST-1 reduction. **b** G6PD inhibitors 6-AN and DHEA attenuated superoxide production in microglia-enriched cultures. **c** Rat mixed-glia cultures grown in black 96-well plates were pretreated with vehicle, 6-AN, or DHEA at the indicated concentration for 30 min prior to the addition of LPS in phenol red-free medium. Fluorescent probe H_2_DCFDA was used to detect inhibition of 6-AN or DHEA on iROS production at 6 h after LPS addition. **d**, **e** At 2 days after LPS treatment, iROS were detected by fluorescent probe CM-H_2_DCFDA (**d**) and quantification of the fluorescent intensity (**e**). **f**, **g** At 2 days after LPS treatment, CM-H_2_DCFDA (green) or anti-4-HNE antibody (red) detected decreases in oxidative stress in microglia-enriched cultures pretreated with 10 μM 6-AN and 100 μM DHEA. Results are mean ± SEM of three to four experiments performed in triplicate (**a**–**c**, **e**, **f**). *p < 0.05 compared with vehicle-treated controls. ^#^*p* < 0.05 compared with LPS-treated cultures

Emerging evidence has indicated that over-activated NOX2, as a major source of oxidative stress under inflammatory condition, plays a pivotal role in chronic neuroinflammation and progressive neurodegeneration [10]. We hypothesized that elevated activity in the PPP increased NADPH production and thereby provided abundant substrate to over-activated NOX2 during neuroinflammation promoting NOX2-derived oxidative stress and subsequent neurodegeneration. To test this hypothesis, we determined whether neuroprotective effects of knockdown and inhibition of G6PD were linked to attenuation of oxidative stress. The results showed that LPS-triggered superoxide release from microglia, measured by SOD-inhibitable reduction of WST-1, was significantly attenuated by siRNA-mediated G6PD knockdown and pharmacological inhibition of G6PD by 6-AN and DHEA (Fig. 3a, b). In addition, pretreatment with 6-AN or DHEA inhibited iROS production in a dose-dependent manner in rat mixed-glia cultures detected by fluorescent probe CM-H_2_DCFDA at 6 h after LPS treatment (Fig. 3c). Similarly, G6PD knockdown in mouse microglia decreased production of iROS at 2 days after LPS treatment (Fig. 3d, e). Moreover, pretreatment of microglia-enriched cultures for 30 min with 6-AN (10 μM) and DHEA (100 μM) significantly attenuated LPS-induced production of iROS and 4-HNE (a major end product of lipid peroxidation and a biomarker for oxidative stress) detected at 2 days after LPS treatment (Fig. 3f, g). LDH and MTT assays detected no cytotoxicity in mouse microglia-enriched cultures treated with 6-AN (10 μM) or DHEA (100 μM) for 1 or 2 days (data not shown). Together, suppression of G6PD activity reduced LPS-elicited oxidative stress.

### Inhibition of PPP activity ameliorated inflammatory response

**Fig. 4:**
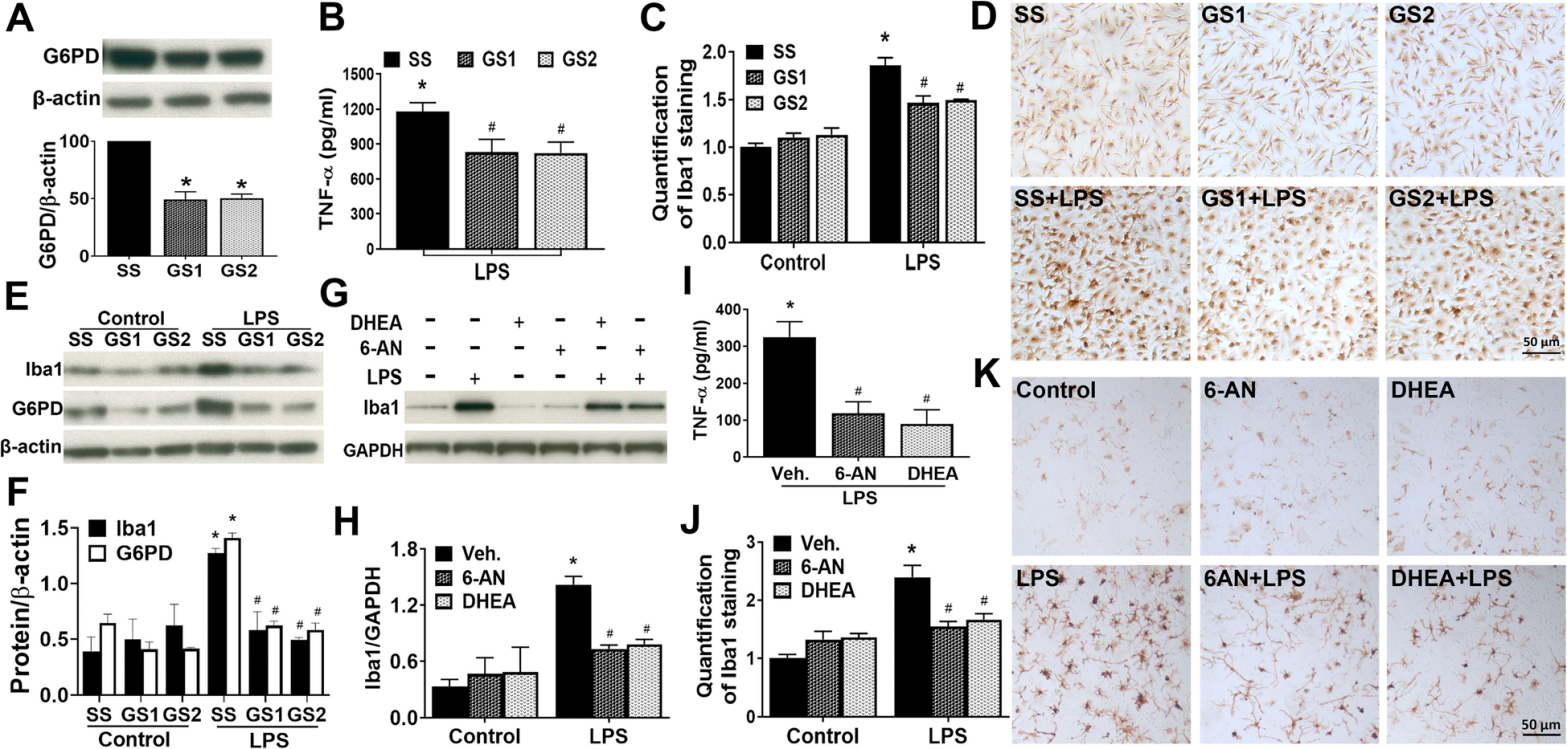
Knockdown or inhibition of microglial G6PD dampened LPS-induced inflammatory response. **a**–**f** At 30 h after transfection with scramble siRNA (SS) and G6PD siRNAs (GS1 and GS2), mouse microglia-enriched cultures were used to examine knockdown efficiency (**a**) or were challenged with vehicle or LPS (**b**–**f**). **a** Significant knockdown of G6PD expression by G6PD siRNAs was detected by Western blotting assay. **b** TNFα secretion was measured by ELISA at 6 h after LPS treatment (the time point for maximal release of TNFα). TNFα levels in vehicle-treated control cultures transfected with SS, GS1, and GS2 were 69.36 ± 51.66, 74.67 ± 44.14, and 72.38 ± 44.44 pg/mL, respectively. **c**–**f** At 18 h after LPS treatment, immunochemistry (**c**, **d**) and Western blotting assay (**e**, **f**) showed significant suppression of microglial activation by G6PD knockdown. **g**–**k** Rat mesencephalic neuron-glia cultures were pretreated with vehicle or G6PD inhibitors 6-AN (10 μM) and DHEA (100 μM) for 30 min prior to the addition of LPS. **g**, **h** Western blotting results indicated that 6-AN and DHEA inhibited LPS-elicited Iba1 upregulation at 2 days after LPS treatment. The ratio of densitometry values of Iba1 normalized to GAPDH in (**g)** was analyzed (**h)**. **i** TNFα secretion was measured by ELISA at 6 h after LPS treatment. TNFα levels in vehicle-treated control cultures with pretreatment with vehicle, 6-AN, and DHEA were 4.64 ± 8.21, 3.68 ± 5.40, and 3.39 ± 5.87 pg/mL, respectively. **j**, **k** The quantification of Iba1 staining and representative immunochemistry images. Results are mean ± SEM of three to four experiments performed in triplicate (**a**–**c** and **f, h**–**j**). *p < 0.05 compared with time-matched vehicle-treated controls. ^#^*p* < 0.05 compared with LPS-treated cultures

Previous studies have shown that peripheral blood mononuclear cells from human subjects with G6PD-deficiency exhibit reduced secretion of inflammatory cytokines such as TNFα and IL-1β [35]. Previous findings including ours indicate that oxidative stress and inflammatory response interact and promote each other [10]. Next, we further investigated whether inhibition of PPP ameliorated LPS-induced inflammatory response and microglial activation. First, detection of knockdown efficiency of G6PD siRNAs in mouse microglia by Western blotting assay showed that the level of G6PD was decreased by ~ 53% after 30-h knockdown by siRNAs (Fig. 4a). Then, at 30 h after transfection with scramble siRNA and G6PD siRNAs, mouse microglia-enriched cultures were challenged with vehicle or LPS and the microglia inflammatory response was evaluated. Both siRNAs of G6PD decreased LPS-induced release of pro-inflammatory cytokine TNFα (Fig. 4b). Morphological evaluation showed that LPS-triggered increases in the number, cell size, and expression of Iba-1 (a specific marker of microglia/macrophages) in microglia were significantly mitigated by G6PD knockdown (Fig. 4c, d). Western blotting assay indicated that while LPS-induced G6PD upregulation was prevented by G6PD siRNAs; in microglia with G6PD knockdown, LPS did not induce Iba1 upregulation (Fig. 4e, f). No cytotoxicity was detected in the cell viability assays (LDH and MTT assays) in microglia-enriched cultures transfected with scramble siRNA and two siRNAs of G6PD for 30 h, which ruled out the possibility that the observed anti-inflammatory effects of G6PD knockdown resulted from cell damage/death caused by transfection process (data not shown). Similarly, LPS-induced inflammatory responses including TNFα release, morphological changes, and Iba1 upregulation in microglia in rat neuron-glia cultures were attenuated by pretreatment with 6-AN (10 μM) and DHEA (100 μM) for 30 min prior to the addition of LPS. Notably, neither 6-AN nor DHEA affected microglial survive or morphology (Fig. 4g–k). Thus, knockdown of G6PD and inhibition of G6PD activity ameliorated inflammatory response.

### Knockdown and inhibition of G6PD suppressed NF-кB activation

**Fig. 5:**
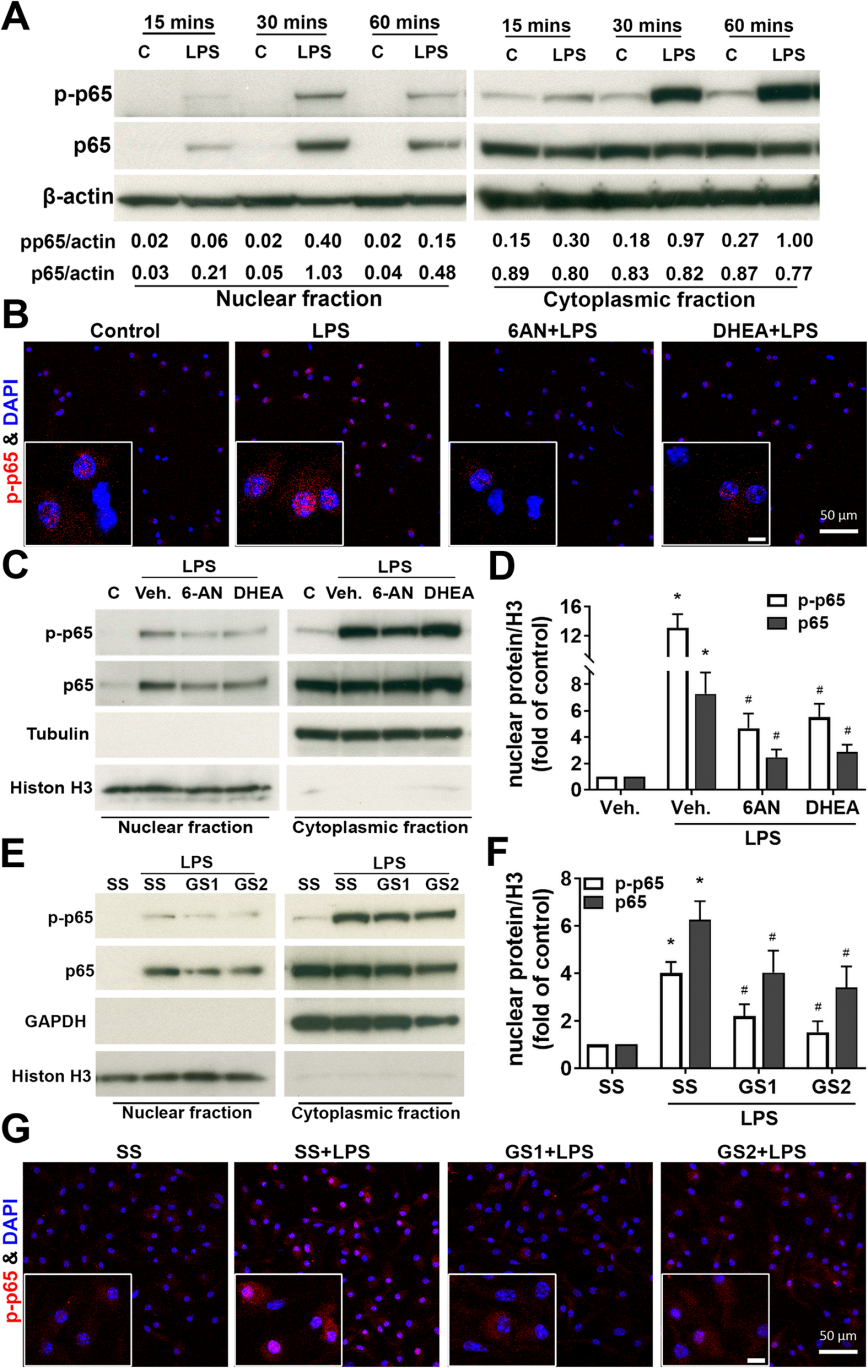
Inhibition or knockdown of G6PD suppressed NF-кB activation. **a** Microglia-enriched cultures were challenged by LPS for 15, 30, and 60 min. The activation of NF-кB pathway, as shown by phosphorylation and nuclear translocation of p65, was observed after LPS treatment. **b**–**d** Microglia-enriched cultures were pretreated with vehicle, 6-AN (10 μM), or DHEA (100 μM) for 30 min prior to the addition of LPS. **b** Immunofluorescence was performed with phosphorylated p65 (red) 30 min after LPS treatment. **c**, **d** Nuclear translocation of p-p65 and p65 was examined after the cultures were treated with 6-AN or DHEA. **e**–**g** At 30 h after transfection with scramble siRNA (SS) and G6PD siRNAs (GS1 and GS2), mouse microglia-enriched cultures were challenged with vehicle or LPS. Nuclear and cytoplasmic proteins were examined at 30 min after LPS treatment to determine the effect of reduced G6PD on NF-кB activation. Results are mean ± SEM of two to three experiments performed in triplicate (**a**, **d**, **f**). *p < 0.05 compared with vehicle-treated controls. ^#^*p* < 0.05 compared with LPS-treated cultures

It is well known that NF-κB participates in cellular responses to stimuli such as stress, cytokines, and free radicals and plays a key role in regulating immune responses. To examine whether suppression of inflammatory responses of microglia by G6PD knockdown or inhibition was mediated by inhibition of NF-κB activation. Activation of the NF-κB pathway, as shown by the phosphorylation and nuclear translocation of p65, was observed after LPS stimulus in microglia-enriched cultures challenged with LPS for 15, 30, and 60 min (Fig 5a). Pretreatment of microglia with 6-AN (10 μM) or DHEA (100 μM) for 30 min attenuated LPS-induced phosphorylation and nuclear translocation of p65 (Fig. 5b–d). Similarly, phosphorylation and nuclear translocation of p65 in LPS-treated microglia-enriched cultures were also prevented by G6PD knockdown (Fig. 5e–g).

### G6PD inhibition by 6-AN injection attenuated LPS-induced oxidative stress, inflammatory response, dopaminergic neurodegeneration, and locomotor impairment

**Fig. 6:**
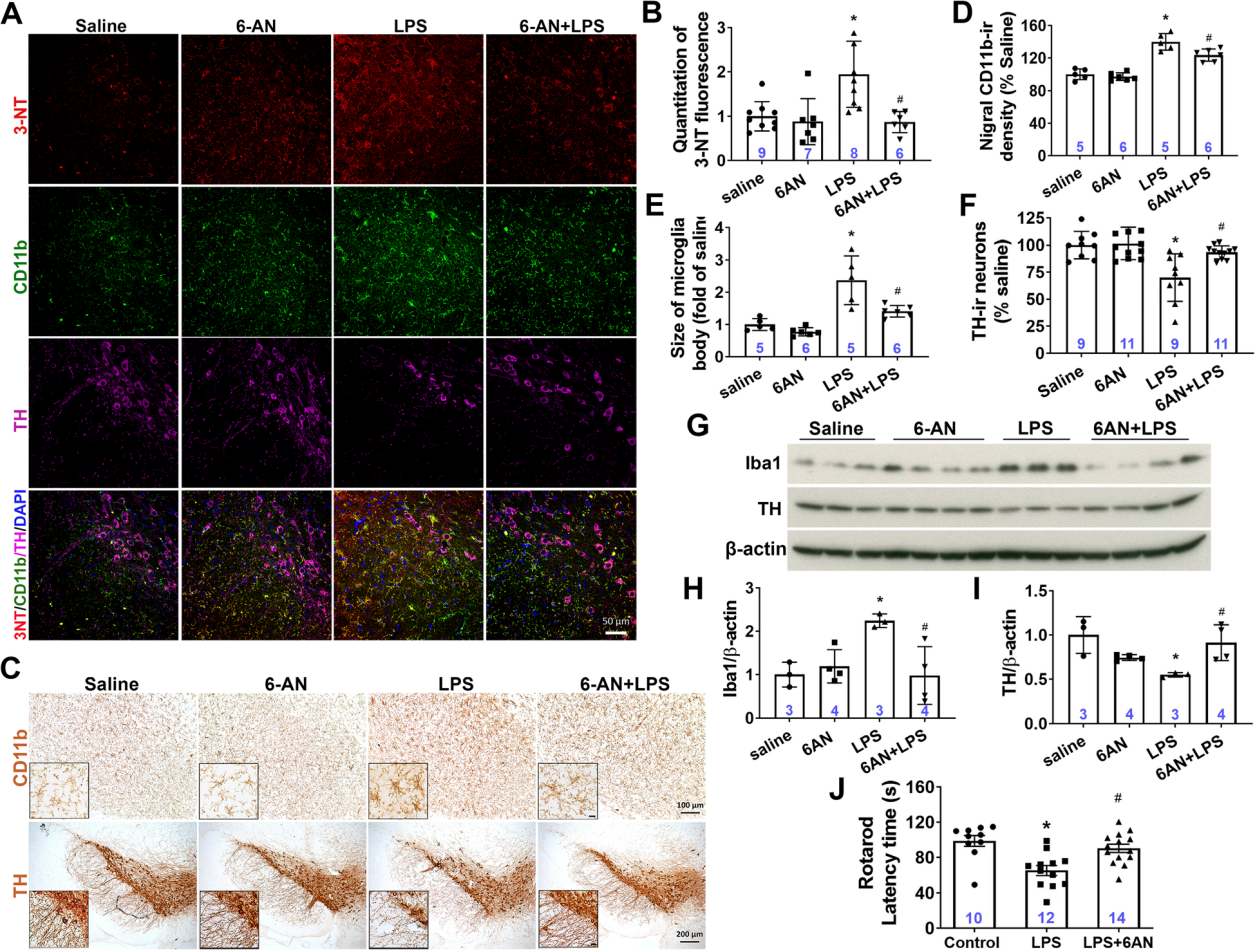
G6PD inhibition attenuated LPS-elicited oxidative stress, microglial activation, DA neurodegeneration, and locomotor deficit. C57BL/6 J mice received an intranigral injection of LPS (2 μg) with 6-AN (20 nmol) or saline. Six weeks later, oxidative stress (**a**, **b**), microglial activation (**a**, **c–e**, **g**, **h**), loss of DA neurons (**a**, **c**, **f**, **g**, **i**), and locomotor deficit (**j**) were determined by immunofluorescence (**a**, **b**), immunochemistry (**c–f**), Western blotting assay (**g**–**i**), and the rotarod behavior test (**j**). **a**, **b** Representative immunofluorescent images (**a**) and the quantification of 3-NT staining showed that injection of 6-AN mitigated LPS-induced oxidative stress in the SN (**b**, saline vs. LPS, *p = 0.0031; LPS vs. 6-AN + LPS, ^#^*p* = 0.0026). **c–e**, **g**, **h** LPS-elicited nigral microglial activation, as shown by enhanced immunoreactivity of CD11b (**d**, saline vs. LPS, *p < 0.0001; LPS vs. 6-AN + LPS, ^#^*p* = 0.009), morphological changes from ramified resting shape to activated appearance with enlarged and irregular cell body (**e**, Saline vs. LPS, *p = 0.0001; LPS vs. 6-AN + LPS, ^#^*p* = 0.0032), the expression of Iba1 (**h**, saline vs. LPS, *p = 0.028; LPS vs. 6-AN + LPS, ^#^*p* = 0.018) was dampened by 6-AN injection. **c**, **f**, **g**, **i** Injection of 6-AN protected DA neurons from LPS-elicited degeneration as shown by improvement in the integrity of TH-IR fibers, the number of TH-IR neurons (**f**, saline vs. LPS, *p = 0.0006; LPS vs. 6-AN + LPS, ^#^*p* = 0.006) and the expression of TH (**i**, saline vs. LPS, *p = 0.016; LPS vs. 6-AN + LPS, ^#^*p* = 0.037). **j** LPS-elicited impairment of locomotor activity of mice measured by the rotarod behavior test was rescued by 6-AN injection (**j**, saline vs. LPS, *p = 0.0006; LPS vs. 6-AN + LPS, ^#^*p* = 0.005). Results are mean ± SEM (**b**, **d**–**f**, **h**–**j**). *p < 0.05 compared with vehicle-injected controls. ^#^*p* < 0.05 compared with LPS-injected groups. Significance was determined by one-way ANOVA with Tukey’s multiple post hoc testing. Immunostaining images were representative of 5 to 11 mice. Numbers within the bars indicate the number of mice in each treatment group

As described above, G6PD knockdown and inhibition attenuated LPS-induced oxidative stress, inflammatory response, and loss of DA neurons in cell culture systems. We next performed intranigral injection of saline or 6-AN (20 nmol) with LPS (2 μg) into C57BL/6 J mice. Considering the nature of DHEA as a steroid hormone and the lack of cell toxicity of 6-AN at doses showing anti-inflammation and neuroprotection in our in vitro studies, we chose to use 6-AN to do the in vivo experiment. At 6 weeks after the injection, oxidative stress, microglial activation, and DA neuron damages were evaluated by immunofluorescence. 3-Nitrotyrosine (3-NT) is thought to be a relatively specific marker of oxidative damage [36] and has high correlation to neurodegenerative diseases [37]. Injection of 6-AN apparently mitigated LPS-induced oxidative stress in the SN (Fig. 6a, b). Increased 3-NT staining was mainly located in microglia and remaining DA neurons (Fig. 6a). Consistent with in vitro findings, in vivo studies also showed that LPS-induced microglial activation in the SN, as shown by CD11b and Iba1 upregulation and switch from ramified resting morphology to activated morphology with enlarged and irregular cell body, was dampened by 6-AN injection (Fig. 6a, c–e, g, h). In addition, nigral DA neurons were protected from LPS-elicited degeneration by 6-AN, as shown by improvement in the number of TH-IR neurons, the expression of TH, and the integrity of TH-IR fibers of the remaining TH-IR neurons in mice co-injected with 6-AN and LPS compared with mice injected with LPS alone (Fig. 6a, c, f, g, i). Moreover, impairment in locomotor activity of mice with LPS injection evaluated by the rotarod behavior test was rescued by 6-AN injection (Fig. 6j). In short, suppression of G6PD activation by intranigral injection of 6-AN attenuated LPS-elicited oxidative stress, microglial activation, dopaminergic neurodegeneration, and locomotor impairment.

## Discussion

The present study has demonstrated that aberrant upregulation of G6PD in microglia exacerbated LPS-induced chronic neurodegeneration through amplifying cellular oxidative stress and inflammatory response. Elevated expression and activity of G6PD were consistently observed in multiple in vitro and in vivo PD models. Knockdown of microglial G6PD and inhibition of G6PD activity by 6-AN and DHEA prevented DA neurons from LPS-induced chronic degeneration. Further mechanistic studies indicated that increased expression and activity of G6PD enhanced NADPH production thereby amplifying LPS-elicited NOX2-derived oxidative stress and NF-κB activation in microglia; these effects were attenuated by knockdown of microglial G6PD and inhibition of G6PD by 6-AN and DHEA. Our findings indicated that G6PD-mediated PPP dysfunction and elevated NADPH production in microglia led to increased production of NOX2-derived ROS amplifying microglial activation and neurodegeneration. Thus, this study has provided the first evidence demonstrating that metabolic disruption in the PPP played a pivotal role in inflammation-induced dopaminergic neurodegeneration.

Studies in animal models and patients have shown glucose metabolism disruption in PD [2, 13, 14]. For instance, PD patients exhibit a decrease in glucose metabolism and abnormal elevation in levels of lactate/pyruvate [38–40]. It is of special interest that several recent studies have revealed dysregulation of the PPP in PD [2, 13]. Transgenic mice with specific overexpression of G6PD in DA neurons are resistant to dopaminergic neurotoxin MPTP [41]. PD-related pesticide paraquat, a superoxide-producing oxidant, increases PPP metabolites (e.g. glucose-6-phosphate and fructose-6-phosphate) and G6PD expression in dopaminergic cell lines, inducing oxidative stress and neurotoxicity that is enhanced by α-synuclein overexpression [42, 43]. Moreover, a postmortem study of PD brains has revealed an increase in NADPH production in the putamen (a brain region affected in PD) but not in the cortex or the cerebellum (unaffected or least affected brain regions in PD) of late-stage cases; unexpectedly, the putamen of early-stage PD and the cerebellum of early- and late-stage PD display a reduction in G6PD [13]. Here, based on regional patterns of Lewy body pathology, PD cases have been grouped into early- and late-stage in which Lewy body pathology is confined to the brainstem and spreads to the limbic and neocortical areas, respectively. However, whether Lewy body pathology accurately reflects the clinical severity and disease progression course of PD is still under debate. In addition, neither NADPH nor G6PD in the SN (the major disease region in PD) has been examined. Earlier measurement of G6PD in red blood cells in PD patients has yielded conflicting results claiming reduced or unaltered activity [20, 21]. The present study detected sustained elevation in expression and activity of G6PD in LPS-treated mesencephalic neuron-glia cultures (an in vitro PD model) and in the SN of four in vivo PD models (Fig. 1). In addition to difference in sample size, examined materials (RBC, leukocyte, neuronal cell lines, human/mouse brain tissues), measurement methods/sensitivity, and disease stage/severity, dynamic changes in the activity of G6PD in different stages of PD might also contribute to the significant heterogeneity among studies [13, 20, 21]. More importantly, inhibition of G6PD and knockdown of microglial G6PD significantly attenuated LPS-elicited dopaminergic neurodegeneration in both in vitro and in vivo models of PD (Figs. 2 and 6). Our findings provided experimental evidence to indicate that G6PD upregulation contributed to PD pathogenesis.

**Fig. 7.**
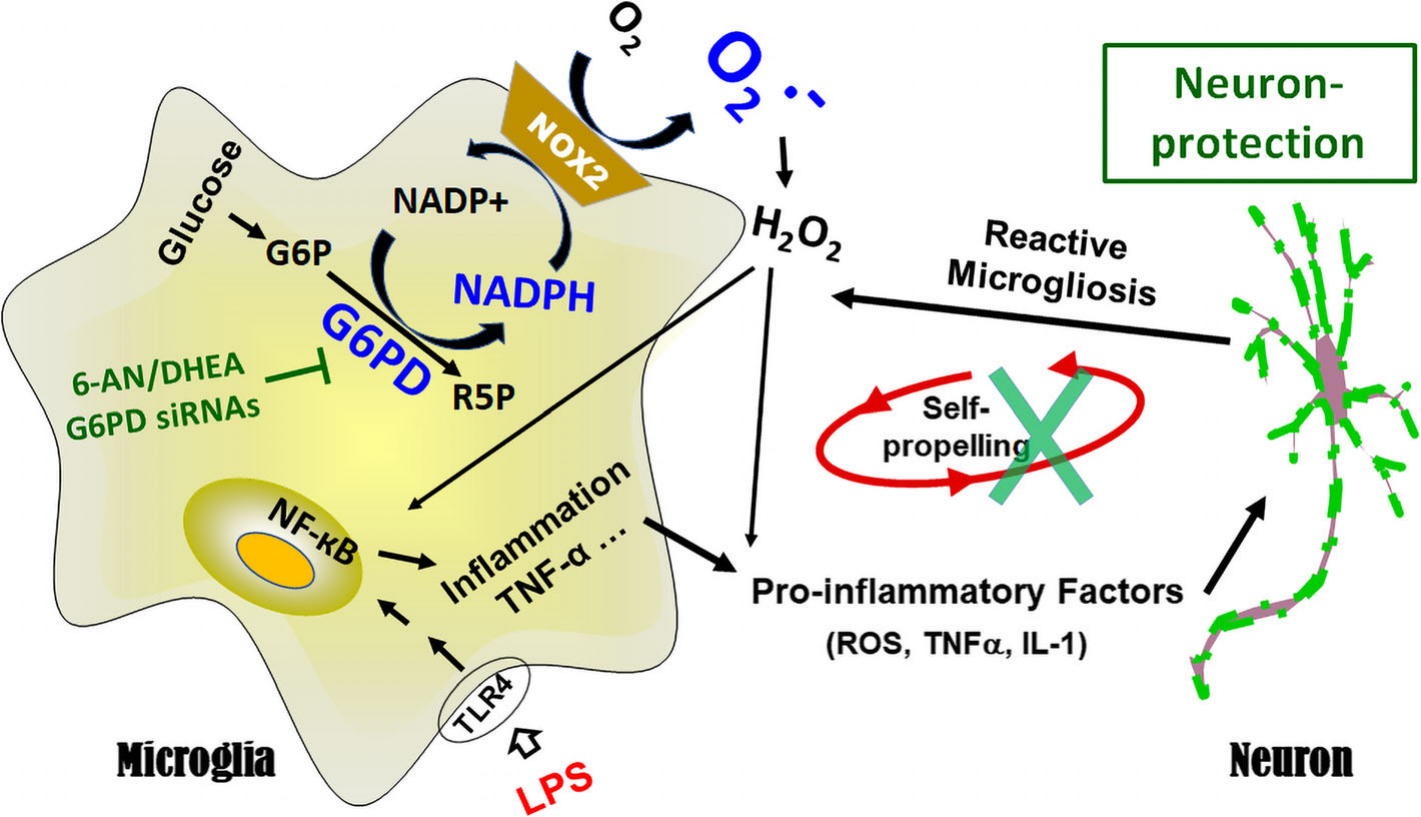
Exacerbation between G6PD dysfunction and neuroinflammation mediated chronic neurodegeneration. The present study elucidated a pathophysiological role of microglial G6PD in PD neurodegeneration. LPS-induced aberrant upregulation of G6PD promoted cellular oxidative stress and NF-кB activation through increasing NADPH availability to NADPH oxidase. Pharmacological inhibition of G6PD activity or biological knockdown of G6PD attenuated oxidative stress, ameliorated inflammatory response of microglia, and prevented DA neurons from LPS-induced chronic degeneration. G6P, glucose-6-phosphate; G6PD, glucose-6-phosphate dehydrogenase; PPP, pentose phosphate pathway; NADPH, nicotinamideadenine-dinucleotide phosphate; NADP^+^, oxidized form of NADPH; R5P, ribose-5-phosphate; LPS: Lipopolysaccharide; 6-AN, 6-aminonicotinamide; DHEA, dehydroepiandrosterone; O_2_^−^, superoxide; H_2_O_2_, hydrogen peroxide; ROS, reactive oxygen species

As one of the three major pathways for the body to generate reducing molecules, the PPP produces approximately 60% of NADPH in humans [44]. NADPH provides the reducing equivalents for biosynthetic/anabolic reactions (e.g. lipid/cholesterol synthesis and fatty acid chain elongation) and oxidation-reduction reactions. NADPH serves as a cofactor of glutathione reductase in generating the reduced glutathione (GSH), one of the most important antioxidants [45–47]. In neurons, NADPH is used as a cofactor for synthesis of fatty acids and myelin, for neurotransmitter turnover, and for redox homeostasis maintenance. NADPH is also required for free radical production by NOX2 and nitric oxide synthase [48]. Thus, NADPH is a central component of both anti- and pro-oxidant processes. The PPP plays an important role in a variety of seemingly diverse human diseases with redox dysregulation as a common denominator, such as hemolytic anemia, autoimmune diseases (e.g. lupus and multiple sclerosis), and male infertility [16]. In some peripheral pathological conditions (e.g. heart failure, atherosclerosis, and obesity), increased activity of G6PD promotes cellular ROS production and pro-inflammatory signaling through increased availability of NADPH to ROS-producing enzymes [32, 49–52]. Furthermore, G6PD upregulation in the heart, liver, and pancreatic β cells of obese and diabetic animals leads to functional defects in the respective tissue by increasing oxidative stress [53–55]. Notably, the SN of patients with PD and mouse models of PD displays upregulation of NOX2 (Fig. 1a, b, e–g) [12]. Over-activated NOX2 is a major source of oxidative stress under inflammatory condition and has been implicated as a novel therapeutic target for neurodegenerative diseases [10]. Microglia with elevated expression and activity of G6PD produced excessive NADPH (Fig. 1) and provided abundant substrate to over-activated NOX2 promoting oxidative stress (Fig. 7).

It is of special interest that G6PD displays high activity in immune cells and participates in the modulation of oxidative stress and inflammatory reactions. An abnormal increase of G6PD in macrophages promotes oxidative stress and inflammatory responses in the adipose tissue of obese mice [56]. G6PD deficiency renders macrophages resistance to LPS stimulation and attenuates insulin resistance in obesity mice through inhibition of adipose tissue inflammation [57]. It has been reported that activated microglia stimulated by LPS with or without interferon γ switch their metabolism from oxidative phosphorylation to glycolysis that also enhances carbon flux to the PPP [58–61]. Knockdown of microglial G6PD or pharmacological inhibition of G6PD activity attenuated LPS-elicited inflammatory responses and oxidative stress (Figs. 3, 4, 5, 6, and 7). Thus, G6PD upregulation and PPP activity elevation in innate immune cells play important roles in chronic glucose metabolism disruption, oxidative stress, and inflammatory responses in both peripheral tissues and the CNS.

Cooperation among different cells in the CNS and optimal balance in the PPP and glycolysis in glucose metabolism must be extremely critical for bioenergetics, redox balance, immune homeostasis, and neuronal survival and should become priority in investigating glucose metabolism dysregulation in brain diseases. Several recent studies have explored cooperation between astrocytes and neurons in glucose metabolism [19, 62, 63]. Disruption of glycolysis in astrocytes and oligodendrocytes triggers axon damage and neurodegeneration [64, 65]. Our findings suggest two-way traffic between metabolic reprogramming and innate immune response in PD pathogenesis. LPS-elicited neuroinflammation triggered abnormal elevation of G6PD expression and activity in microglia, which in turn exacerbated neuroinflammation thereby inducing dopaminergic neurodegeneration. Oxidative stress is the shared predominant mediator during this pathogenic process. Indeed, it has recently been proposed that metabolic-inflammatory axis plays important roles in brain aging and neurodegeneration [22].

Multiple lines of evidence including ours from the present study reveal important contribution of G6PD/PPP dysfunction and consequent oxidative stress to PD pathogenesis and suggest that modulation of G6PD activity might be beneficial for PD treatment. However, caution is needed on targeting G6PD and the PPP as a therapeutic intervention, because neurons greatly rely on the PPP to generate NADPH and its downstream antioxidant GSH to maintain their redox homeostasis, integrity, and normal function. Unlike most other cell types, neurons preferentially metabolize glucose via the PPP because constant ubiquitination and proteasomal degradation of phosphofructokinase B3, the rate-limiting enzyme and master regulator of glycolysis [19]. G6PD deficiency, an X-linked recessive genetic disorder, is the most common enzymopathy affecting approximately 400 million people worldwide. The most common clinical manifestation associated with G6PD deficiency is hemolytic anemia [66]. Acute hemolysis associated with G6PD deficiency usually is short-lived and self-limited. G6PD deficiency is a manageable disease that requires strict avoidance of oxidative stressor in the form of infection, oxidative drugs, or foods (e.g. fava beans). These features of G6PD suggest that it is feasible to treat PD through dynamic modulating G6PD activity and to avoid compromising RBCs, neurons, the immune system, and other physiological function.

## Conclusions

Overall, our data demonstrated, for the first time, that G6PD and the PPP played a pivotal role in LPS-induced degeneration of DA neurons and locomotor impairment. Metabolic disruption in the PPP and neuroinflammation exacerbated each other inducing PD-like neurodegeneration and locomotor impairment. Results of this study shed light on metabolic-inflammatory mechanisms of PD pathogenesis and suggest that modulation of activity of G6PD and NOX2 may become potential therapeutic interventions in PD.

## Supplementary information

**Additional file 1: Figure S1.** The pattern of G6PD expression in microglia-containing cultures. (**A**) Immunocytochemical staining for G6PD on neuron-glia cultures (left panel) and microglia-enriched cultures (middle panel) and double-labeled immunofluorescent staining on microglia-enriched cultures (right panel) detected a significant increase in the level of G6PD protein at 7 days and 24 h after LPS treatment for neuron-glia cultures and microglia-enriched cultures respectively. (**B**) Densitometric measurements of G6PD immunoreactivity in (**A**).

## Data Availability

The datasets used and/or analyzed during the current study are available from the corresponding author on reasonable request.

## Abbreviations

3-NT: 3-Nitrotyrosine
4-HNE: 4-Hydroxynonenal
6-AN: 6-Aminonicotinamide
CM-H_2_DCFDA: Chloromethyl-29, 79-dichlorodihydrofluorescein diacetate
CNS: Central nervous system
DA: Dopamine
DHEA: Dehydroepiandrosterone
FBS: Fetal bovine serum
G6PD: Glucose-6-phosphate dehydrogenase
GAPDH: Glyceraldehyde 3-phosphate dehydrogenase
GFAP: Glial fibrillary acidic protein
GS1/GS2: G6PD siRNAs
GSH: Reduced glutathione
HLA: Human leukocyte antigen
HS: Horse serum
Iba1: Ionized calcium-binding adaptor molecule-1
IL-1β: Interleukin 1 beta
iROS: Intracellular reactive oxygen species
LDH: Lactate dehydrogenase
LME: Leucine-leucine methyl ester
LPS: Lipopolysaccharide
MEM: Minimum essential medium
MPTP: 1-Methyl-4-phenyl-1,2,3,6-tetrahydropyridine
MTT: Thiazolyl blue tetrazolium bromide
NADPH: Nicotinamide adenine dinucleotide phosphate
Neu-N: Neuron-specific nuclear protein
NF-кB: Nuclear factor kappa B
NOX2: NADPH oxidase
PD: Parkinson’s disease
PPP: Pentose phosphate pathway
ROS: Reactive oxygen species
SN: Substantia nigra
SOD: Superoxide dismutase
SS: Scramble siRNA
TH-IR: Tyrosine hydroxylase-immunoreactive
TNFα: Tumor necrosis factor-α
WST-1: Water-soluble tetrazolium salt 1
