# Supplementary figures and images for: The pentose phosphate pathway regulates chronic neuroinflammation and dopaminergic neurodegeneration
Source: J Neuroinflammation. 2019 Dec 5;16:255. doi: 10.1186/s12974-019-1659-1 (PMC6896486; doi:10.1186/s12974-019-1659-1)

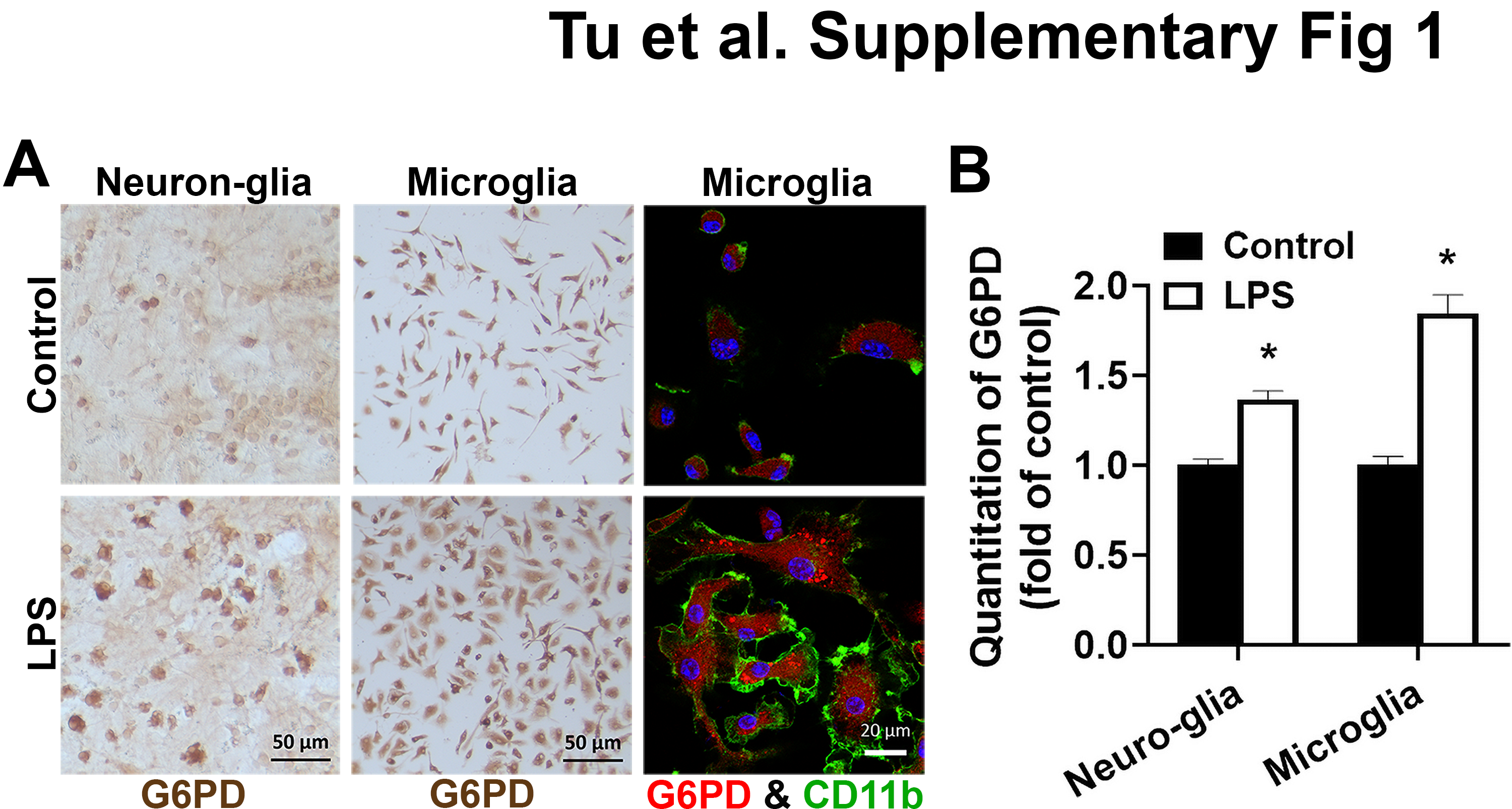

Supplement: Supplementary file 1 — Additional file 1: Figure S1. The pattern of G6PD expression in microglia-containing cultures. (A) Immunocytochemical staining for G6PD on neuron-glia cultures (left panel) and microglia-enriched cultures (middle panel) and double-labeled immunofluorescent staining on microglia-enriched cultures (right panel) detected a significant increase in the level of G6PD protein at 7 days and 24 h after LPS treatment for neuron-glia cultures and microglia-enriched cultures respectively. (B) Densitometric measurements of G6PD immunoreactivity in (A). [file 12974_2019_1659_MOESM1_ESM.tif]
